# Prevalence and Related Factors for Myopia in School-Aged Children in Qingdao

**DOI:** 10.1155/2018/9781987

**Published:** 2018-01-08

**Authors:** Jin Tao Sun, Meng An, Xiao Bo Yan, Guo Hua Li, Da Bo Wang

**Affiliations:** ^1^Department of Ophthalmology, Qingdao Economic and Technological Development Area First People's Hospital, Qingdao, Shandong 266555, China; ^2^Department of Ophthalmology, Qingdao Traditional Chinese Medical Hospital of Huangdao District, Qingdao, Shandong 266500, China; ^3^Department of Ophthalmology, The Affiliated Hospital of Qingdao University, Qingdao, Shandong 266000, China

## Abstract

**Purpose:**

To investigate the prevalence and related factors for myopia in school-aged children in the Economic and Technological Development Zone of Qingdao, Eastern China.

**Methods:**

A total of 4890 (aged 10 to 15 years) students were initially enrolled in this study. 3753 (76.75%) students with completed refractive error and questionnaire data were analyzed. The children underwent a comprehensive eye examination. Multiple logistic regression models were applied to assess possible factors associated with myopia.

**Results:**

The prevalence of myopia increased as the children's grade increased (*χ*^2^ = 560.584, *P* < 0.001). Low myopia was the main form of myopia in adolescent students (30.22%). With the growth of age, students spent significantly more time on near work (*P* = 0.03) and less time on outdoor activity (*P* < 0.001). In multivariate models, only the following variables were significantly associated with myopia: age, two myopic parents, outdoor activity time, and continuous near work without 5 min rest.

**Conclusions:**

The prevalence of myopia increased as the grade increased. Age, two myopic parents, and continuous near work time without 5 min rest were risk factors for myopia. Outdoor activities had protective effect for myopia.

## 1. Introduction

Myopia has become a major global public health problem, particularly in East Asia [1]. The direct cost of providing eyeglasses to people who need refractive correction are also enormous. In the United States, the National Health and Nutrition Examination Survey (NHANES) reported the annual direct cost of correcting distance vision impairment due to refractive errors to be between US$3.9 and US$7.2 billion [2]. The prevalence of myopia is generally highest in populations of East Asian, particularly in urban locations such as Guangzhou [3], Taiwan [4], Hong Kong [5], and Korea [6]. Affected by many factors, such as visual function, psychology, aesthetics, and economy, the quality of life in patients of myopia was seriously impaired [7].

In recent years, Chinese scientific research institutions have carried out large-scale epidemiological survey on myopia in the northern and southern areas. Numerous cross-sectional studies have provided information on the pattern of prevalence and risk factors for myopia. Although the exact pathogenic mechanism of myopia is still unclear, most scholars believe that myopia is the result of a combination of genetic and environmental factors. Recent epidemiological surveys have shown that the prevalence of myopia varies widely, depending on age, gender, geography, and ethnicity [8–11]. What is more, some studies have explored other relevant influencing factors, including more time spent on near work activity [12], higher educational level [13], and less time participating in outdoor activities [14]. Whereas the evidence on this issue is controversial, a cross-sectional study in Beijing demonstrated that a higher prevalence of myopia in high school students was associated with shorter near work distance [15]. Lin et al. [16] reported that outdoor activities were associated with less myopic refraction, but they did not find any significant association between near work and myopic refraction in this study. Furthermore, Low et al. [17] reported that neither near work nor outdoor activity was found to be associated with early myopia. The conflict results are mainly attributed to the following aspects: (1) There are no uniform questionnaire. (2) The results of questionnaire survey are affected by geography, culture, cognitive ability, and memory biases of the respondents. (3) The outcome used to reflect myopia was mainly noncycloplegic autorefraction. Recently, a standardized myopia questionnaire, which was developed by the Sydney Myopia Study group, was used to acquire information on near work/outdoor activities, habitual reading distance, and so on [18]. In this study, we investigated the prevalence and the risk factors for myopia in schoolchildren in the eastern coastal city of China, by the method of cluster sampling, with particular attention to variables such as the duration and type of outdoor activities and near work.

## 2. Methods

### 2.1. Study Participants

The Childhood Errors of Refraction Study was a cross-sectional epidemiological study investigating the prevalence of refractive error in 10–15-year-old school-aged children conducted from December 2015 to January 2016. The sample calculation formula *n* = (*μα*/*δ*)^2^*p*(1 − *p*) was used to estimate the number of samples. With a stratified-clustered sampling method, 6 primary (aged 7 to 12 years) and 4 secondary (aged 13 to 15 years) schools including 4890 students (2529 [51.72%] male) were randomly selected from 22 primary and 19 secondary schools. This met the sample number criteria for total number of samples, providing a representative sample of Economic and Technological Development Zone of Qingdao primary and secondary schools.

### 2.2. Ethics Statement

The study was approved by the Ethics Committee of the Review Board of the Qingdao Economic and Technological Development Area First people's Hospital and adhered to the Declaration of Helsinki. Written informed consent was obtained from parents or guardians.

### 2.3. Examination

The children underwent a comprehensive eye examination, including measurement of visual acuity, color vision, assessment of ocular motility, slit-lamp examination, autorefraction, cycloplegic autorefraction, and fundus examination using a direct ophthalmoscope (YZ6E; Six Six Vision Corp., Suzhou, China). The cycloplegic autorefraction was measured by a binocular open-field autorefractor (RM-8000A, Topcon, Japan) with a measurement range of −25 to +22 diopters (D). Cycloplegia was induced in each eye by instillation of three drops of 0.5% tropicamide 5 min apart. Extra tropicamide (1 or 2 drops) was also used in some children to obtain adequate mydriasis (a minimum pupil diameter of 6 mm and disappearance of papillary light reflex).

### 2.4. Questionnaire Survey

A standardized myopia questionnaire, which was modified from the Sydney Myopia Study (SMS) group, was adapted and applied to this study. The questionnaire was translated by ophthalmologists, an epidemiologist, and a statistician in our study group. It is composed of two parts: the parental version and the children's version. A pilot study in the Anyang Childhood Eye Study (ACES) proved that this questionnaire is valid and reliable [19]. In order to ensure the quality of investigation, school mobilization was implemented in selected school sampling units by project members through a meeting in which the details regarding the questionnaire were explained to the parents and guardians. Primary school students were allowed to complete the questionnaire with the help of parents. The questionnaire was administered to obtain information on near work time, continuous near work time without 5 min rest, near work distance, outdoor activities, and so on. Parental refractive status was also obtained from the questionnaire. Average hours spent on near work (<50 cm working distance) were summed from questions regarding drawing, homework, reading, making handicrafts, and handheld computer use. Time spent on outdoor activities was based on questions about playing outdoors, family picnics, taking a walk, bicycle riding, hiking, and outdoor sports after school on weekdays and weekends. To assess the duration of continuous reading, children were asked about the time they spent in continuous reading or other near work before taking a break of 5 minutes or longer. They were then classified into five categories: category A: 0–15 min; category B: 15–30 min; category C: 30–45 min; category D: 45–60 min; and category E: >60 min. From response to a question “How far did you often write your homework,” the distance from objects when doing near work was classified into four categories: category A: >30 cm; category B: 20–30 cm; category C: 10–20 cm; and category D: <10 cm.

### 2.5. Quality Control

The equipment was checked and calibrated daily. All examiners were senior clinical ophthalmologists. Data entry was completed by well-trained staff.

### 2.6. Definitions and Data Analysis

Spherical equivalent (SE) was calculated with the following equation: SE = spherical diopter +0.5 × cylinder diopter. Myopia, emmetropia, and hyperopia was defined as the SE < −0.50 D (low myopia < −0.5 to >−3.0 D, moderate myopia ≤ −3.0 to >−6.0 D, and high myopia ≤ −6.0 D), −0.50 D ≤ SE ≤ +0.50 D, and SE > + 0.50 D, respectively [20]. Statistical analysis was performed using a commercially available statistical software package (SPSS for Windows, version 20.0, IBM-SPSS, Chicago, Illinois, USA). First, we examined the associations between the prevalence of myopia and other parameters in a univariate analysis. Multiple logistic regression analysis was then used to determine independent factors. Odds ratios (OR) and their 95% confidence intervals (CI) for myopia were calculated. All *P* values were 2-sided and were considered statistically significant when the values were less than 0.05.

## 3. Result

The mean refractive error was −1.62 (±1.82) D, and the overall prevalence of myopia was 52.02%. The prevalence of myopia in students increased with age (*χ*^2^ = 560.58, *P* < 0.001); the prevalence of myopia in students at 10 years old was only 22.61%, as it increased to 56.93% in students at 13 years old, and the rate was the highest (69.34%) in students at 15 years old (Table 1). There was no significant statistical difference in prevalence of myopia between boys and girls (*χ*^2^ = 0.709, *P* = 0.400, Table 2).

In addition, we found that low myopia was still the main form of adolescent myopia. The proportion of high myopia increased with age (*χ*^2^ = 567.054, *P* < 0.001, Table 3).  Table 4 presents the time that students spent on near work and outdoor activities. The older children had spent significantly more time on near work (*P* = 0.03) and less time on outdoor activities than the young ones (*P* < 0.001).

The results of univariate and multivariate analyses of factors associated with myopia are shown in Table 5. Univariate analysis showed that the following variables were significantly associated with myopia: age, one myopic parent, two myopic parents, near work distance, near work time, outdoor activity time, and continuous near work without 5 min rest. In multivariate models, only the following variables were significantly associated with myopia: age, two myopic parents, outdoor activity time, and continuous near work without 5 min rest.

## 4. Discussion

The prevalence of myopia around the world has increased recently. Previous studies have shown that 9 to 16 years of age is the fastest growing period for adolescent myopia [21]. Other than genetic factors, environment is also an important contributing factor in the development of myopia [22]. Scholars from all over the world have done a lot of research on the environmental factors, but the specific mechanism and extent of this impact remain controversial.

Consistent with previous studies, we found that the prevalence of myopia in students persistently increased as the age increased. Interestingly, this result is lower than that in urban areas in Guangzhou [23], which is higher than that in rural areas in Yangxi [24]. In addition, low myopia is the main form of myopia, but the properation of high myopia increased as the age increased. We consider that this might be related to the social and economic environment in this region. From another point of view, the importance of environmental factors for myopia is explained.

At present, there is no unified conclusion about the prevalence of myopia among male or female. The current results revealed that girls were no more likely to suffer from myopia than boys. This is consistent with many previous studies [8, 25]. Particularly, in the COMET study, although there is no difference in the prevalence of myopia between boys and girls, boys had a slower progression (by 0.16 D) than girls [26]. They considered that any relationship with sex, if it existed, would occur early in the course of myopia and would not be sustained over time. We think that this explanation may be reasonable. Taking into account the age group of the participants in the study, we believe that this explanation is reasonable.

Previous studies showed that parental myopia, in even only one parent, leads to an increased risk for juvenile myopia. In Australia, in six-year-old children, there was 3.16- and 3.33-fold increased risk of incident myopia than no parental myopia, respectively [25]. One interesting finding of this study was that although one or two parental myopia was a risk factor for myopia in univariate analysis, only two parental myopia was a risk factor after multiple regression analysis. This result may provide us with some valuable information about the relationship between heredity and myopia.

Previous numerous cross-sectional studies had reported that schoolchildren engaged in near work were more likely to have myopia than those who spent less time on near work [27, 28] and whose distance of near work were shorter than 30 cm [29, 30]. However, there were also some studies that have reported lack of association between near work and myopia [31, 32]. Thus, the findings are equivocal. In this study, with the growth of age, students spent significantly more time on near work than before. The near work time increased from 3.32 h/d in the 10-year-old children to 4.62 h/d in the 15-year-old children. However, after multiple regression analysis, we found that near work time and near work distance were not significantly related to myopia. Perhaps, as Lin et al. [16] assumed, there was a special “saturation effect” between them.

Consistent with previous study [33], we found that children whose continuous near work time > 30 min without 5 min rest were more likely to have myopia than those 0–15 min group. Perhaps, we could put forward such a hypothesis that there was a “dose reponse” between myopia and the duration of continuous near work. In other words, as long as near work time reached a certain intensity, it would have an impact on myopia, which meaned that the intensity of near work rather than the total time was an important factor for myopia. However, it should be pointed out that some scholars considered that there was a positive association between a higher education level and myopia [34]. However, we thought that a higher academic level was highly correlated with near work time and it should not be listed separately.

In Singapore, a cross-sectional study was conducted to analyze the effect of outdoor activities on 1249 teenagers aged 11–20 years. They found a significant negative association between myopia and outdoor activities. Adjusting for the confounders, for each hour increase in outdoor activities per day, SE increased by 0.17 D and the axial length decreased by 0.06 mm [35]. Some scholars had also come up with the quantitative standard of outdoor activity time. Jones et al. [36] found that there might be a threshold of around 2-3 hours per day spent outdoors that was needed to prevent myopia. Smith et al. [37] found that high ambient lighting retarded the development of experimental myopia in monkeys. The possible explanations included that high ambient lighting could regulate the release of dopamine from the retina and stimulate the synthesis of vitamin D in the body [38, 39]. In the present study, the outdoor activity time decreased from 2.28 h/d in the 10-year-old children to 1.42 h/d in the 15-year-old children. Similar to previous studies, we found that the more time spent outdoors was associated with a lower prevalence of myopia. Although the specific mechanism remained to be further studied, the increase of outdoor activities as an effective method of preventing myopia was worth recommending.

In addition, it should be particularly pointed out that the questionnaire used in this study was similar to that of ACES. As the latest study on children myopia in China, the Anyang Childhood Eye Study has completed a series of horizontal and longitudinal studies on myopia. Therefore, we made a comparison of the two studies. At age 12 years, our children had similar level of near work time (3.41 versus 3.70 h/d) and outdoor activities time (2.06 versus 2.08 h/d) with the Anyang cohort [40–42]. Therefore, we have reason to believe that the data of this survey are worthy of belief. Note that our children at age 12 years had significantly lower myopic prevalence (45.75% versus 67.3%) than theirs. These differences could not be explained by the time in near work and outdoor activities. By comparing with the Sydney Myopia Study, Li et al. [41] found a similar problem. They led to the idea that some behaviors during near work were more likely to play an important role in myopia. We thought that this idea was reasonable. We will make a further comparison of these related parameters between them in the following study.

Although there are several important findings in our study, the results of our analyses were tempered by some limitations. First, the data about near work, outdoor activities, and its related parameters was obtained from questionnaires. Although this method was predominant in previously reported studies, it could be subjected to recall bias. Second, the whole cycloplegic autorefraction data collection process lasted about 2 months, so there might be measurement bias. Third, there are some examples of using tropicamide for cycloplegia, but there are more international research examples of using cyclopentolate in recent years. As we all know, tropicamide is not as strong as cyclopentolate for paralyzing ciliary muscle. We finally chose tropicamide as cycloplegic agent mainly because we found some parents worried about the possible or potential side effects, and they also worry about that mydriasis for three days will affect children's learning. If cyclopentolate is used, majority of parents will refuse to attend the study. Therefore, there may be some errors in the results of cycloplegic autorefraction. Lastly, this was only a cross-sectional survey; thus, we could not draw any conclusion about the incidence and progression for myopia.

In conclusion, the prevalence of myopia in adolescent students increased as the grade increased. Age, two myopic parents, and continuous near work time without 5 min rest were risk factors for myopia. Longer time spent on outdoor activities was significantly associated with a lower risk of myopia. These associations may indicate that low intensity near work and more outdoor activities may be important for future trials of intervention on myopia.

## Figures and Tables

**Table 1:** The prevalence of myopia in different age groups.

Age (years)	Number (*n*)	Myopia (*n*)	Myopia (%)
10	690	156	22.61
11	678	222	32.74
12	671	307	45.75
13	685	390	56.93
14	1080	716	66.30
15	1086	753	69.34
Total	4890	2544	52.02

**Table 2:** The prevalence of myopia of different genders in different age groups.

Age (years)	Male		Female	
	*n*	Myopia (%)	*n*	Myopia (%)
10	361	94 (26.0)	329	62 (18.8)
11	348	119 (34.2)	330	103 (31.2)
12	351	156 (44.4)	320	151 (47.2)
13	344	199 (57.8)	341	191 (56.0)
14	560	346 (61.8)	520	370 (71.2)
15	565	387 (68.5)	521	366 (70.2)
Total	2529	1301 (51.4)	2361	1243 (52.6)

**Table 3:** The prevalence of low, moderate, and high myopia in different age groups.

Age (years)	No myopia		Low myopia		Moderate myopia		High myopia	
	*n*	%	*n*	%	*n*	%	*n*	%
10	534	77.39	130	18.84	17	2.46	9	1.30
11	456	67.26	162	23.89	50	7.37	10	1.47
12	364	54.25	192	28.61	89	13.26	26	3.87
13	295	45.07	226	32.99	117	17.08	47	6.86
14	364	33.70	380	35.19	247	22.87	89	8.24
15	333	30.66	388	35.73	266	24.49	99	9.12
Total	2346	47.98	1478	30.22	786	16.07	280	5.73

**Table 4:** Near work and outdoor activity time (hours per day) of the students.

Age (years)	*n*	Near work (h/d)	Outdoor activity (h/d)
		(Mean ± SD)	(Mean ± SD)
10	562	3.32 ± 1.32	2.28 ± 1.21
11	559	3.42 ± 1.56	2.24 ± 1.26
12	536	3.41 ± 1.82	2.06 ± 1.32
13	561	3.78 ± 1.42	1.88 ± 1.12
14	752	4.32 ± 1.84	1.64 ± 1.14
15	783	4.62 ± 1.26	1.42 ± 0.96
*P* value		*P* = 0.03	*P* < 0.001

**Table 5:** Associations between myopia and possible risk factors.

Variables	Univariate analysis			Multivariate analysis		
	Odds ratio	95% CI	*P*	Odds ratio	95% CI	*P*
Age	1.43	1.34–1.52	0.012^∗^	1.23	1.18–1.27	<0.001^∗^
Sex						
Boys	1			1		
Girls	1.64	0.48–2.21	0.14	1.68	0.42–1.92	0.12
Parental myopia						
None	1			1		
One myopic	1.47	1.24–1.96	0.01^∗^	1.62	0.71–2.34	0.12
Two myopic	2.32	1.72–3.28	<0.001^∗^	2.58	1.76–3.46	<0.001^∗^
Near work distance (cm)						
>30	1			1		
20–30	1.27	1.02–1.54	<0.001^∗^	1.12	0.69–1.38	0.23
10–20	2.46	1.52–4.76	<0.001^∗^	1.76	0.49–2.74	0.18
0–10	1.29	1.08–1.54	0.04	1.21	0.84–1.41	0.32
Trend test			0.16			0.21
Near work time (h/d)	1.28	1.04–1.86	<0.001^∗^	1.42	0.79–2.04	0.16
Outdoor activity time (h/d)	0.67	0.46–0.78	0.03^∗^	0.74	0.53–0.92	<0.001^∗^
5 min rest after continuous near work time (min)						
0–15	1			1		
15–30	0.94	0.72–1.12	0.24	1.02	0.92–1.08	0.12
30–45	1.19	1.02–1.31	0.02^∗^	1.24	1.14–1.32	<0.001^∗^
45–60	1.36	1.12–1.49	<0.001^∗^	1.34	1.28–1.38	0.03^∗^
>60	2.12	1.76–2.72	<0.001^∗^	2.48	1.92–3.24	<0.001^∗^
Trend test			<0.001^∗^			<0.001^∗^

∗ indicates a significant statistical significance (*P* < 0.05).
